# Magnetic resonance imaging findings for discriminating clear cell carcinoma and endometrioid carcinoma of the ovary

**DOI:** 10.1186/s13048-019-0497-1

**Published:** 2019-02-25

**Authors:** Sachiko Morioka, Ryuji Kawaguchi, Yuki Yamada, Kana Iwai, Chiharu Yoshimoto, Hiroshi Kobayashi

**Affiliations:** 1 Department of Obstetrics and Gynecology, Nara Medical University, Shijo-cho 840, Kashihara, Nara, 634-8522 Japan; 2 Department of Obstetrics and Gynecology, Yao Municipal Hospital, 1-3-1 Ryuge-cho, Yao, Osaka, 581-0069 Japan

**Keywords:** Carcinoma, Endometrioid, Endometriosis, Logistic Models, Ascites, Pathology, Surgical, Adenocarcinoma, Clear Cell, Multivariate Analysis, Magnetic Resonance Imaging

## Abstract

**Background:**

Common cancerous histological types associated with endometriosis are clear cell carcinoma (CCC) and endometrioid carcinoma (EC). CCC is regarded as an aggressive, chemoresistant histological subtype. Magnetic resonance imaging (MRI) offers some potential advantages to diagnose ovarian tumors compared with ultrasonography or computed tomography. This study aimed to identify MRI features that can be used to differentiate between CCC and EC.

**Methods:**

We searched medical records of patients with ovarian cancers who underwent surgical treatment at Nara Medical University Hospital between January 2008 and September 2018; we identified 98 patients with CCC or EC who had undergone preoperative MRI. Contrasted MRI scans were performed less than 2 months before surgery. Patients were excluded from the study if they had no pathology, other pathological subtype of epithelial ovarian cancer, and/or salvage treatment for recurrence and metastatic ovarian cancer at the time of study initiation. Clinically relevant variables that were statistically significant by univariate analysis were selected for subsequent multivariate regression analysis to identify independent factors to distinguish CCC from EC.

**Results:**

MRI of CCC and EC showed a large cystic heterogeneous mixed mass with mural nodules protruding into the cystic space. Univariate logistic regression analysis revealed that the growth pattern (broad-based nodular structures [multifocal/concentric sign] or polypoid structures [focal/eccentric sign]), surface irregularity (a smooth/regular surface or a rough/irregular/lobulated surface), “Width” of mural nodule, “Height-to-Width” ratio (HWR), and presence of preoperative ascites were factors that significantly differed between CCC and EC. In the multivariate logistic regression analysis, the growth pattern of the mural nodule (odds ratio [OR] = 0.69, 95% confidence interval [CI]: 0.013–0.273, *p* = 0.0004) and the HWR (OR = 3.71, 95% CI: 1.128–13.438, *p* = 0.036) were independent predictors to distinguish CCC from EC.

**Conclusions:**

In conclusion, MRI data showed that the growth pattern of mural nodules and the HWR were independent factors that could allow differentiation between CCC and EC. This finding may be helpful to predict patient prognosis before operation.

## Background

Ultrasonography, computed tomography (CT), and magnetic resonance imaging (MRI) have been established as useful tools in the multimodality approach to detect, characterize, and diagnose ovarian tumors [1]. Ultrasonography is advantageous, compared with CT and MRI, due to its accessibility as a first-line imaging examination, which is painless and relatively inexpensive compared with CT and MRI. However, MRI offers the following potential advantages compared with the other modalities: lack of ionizing radiation exposure relative to CT, higher contrast resolution, higher specificity, greater accuracy, more reliable and reproducible measurements, and good inter-observer agreement for identification of malignant ovarian lesions [2]. The accuracies of ovarian cancer patient diagnosis with ultrasonography, CT, and MRI are 84–89%, 82–88%, and 88–93%, respectively [3–7].

Several studies have investigated the diagnostic value of various MRI sequences, including T1-weighted, T2-weighted, T1-weighted with fat suppression and contrast, diffusion-weighted (DW) MRI, and dynamic contrast-enhanced MRI, in the evaluation of benign and malignant ovarian lesions [8–10]. Characteristic features of epithelial ovarian cancer include the presence of the following: a cystic mixed mass (complex solid and cystic), varying proportions of a solid-enhancing component, a mural nodule or papillary projection and internal thick septation, central necrosis, tumor vascularity, ascites, peritoneal implants, and lymph node enlargement [10, 11].

Epithelial ovarian cancers are classified as one of two types: type I includes patients with low-grade serous carcinoma, low-grade endometrioid carcinoma (EC), clear cell carcinomas (CCC), mucinous carcinoma, Brenner tumors, and slow-growing tumors; and type II includes patients with rapidly growing high-grade serous carcinoma (HGSC), high-grade endometrioid carcinoma, undifferentiated carcinomas, and highly aggressive malignancies [12]. CCC and EC are the most common types of ovarian cancer highly associated with endometriosis [13]. The size of ovarian endometrioma (tumor size ≥9 cm), multiple foci of endometriosis, and presence of tumors with solid components or mural nodules, are risk factors that are likely associated with malignant transformation of endometriosis [14–16]. CCC is the second most common histologic subtype (27.6%) of epithelial ovarian cancer in Japan [17, 18]. Compared with its incidence in Caucasians, CCC is more common in Asians [19]. There is a substantial difference between type I and type II epithelial ovarian cancers in terms of the MRI morphological features [20, 21]. The imaging characteristics of type I tumors frequently include the presence of a predominantly cystic mass, a larger lesion, mural nodules, papillary architecture, and strong enhancement, relative to those aspects in type II tumors [10].

It is important to distinguish between CCC and EC because CCC is associated with a poor prognosis, based on its chemoresistant phenotype. However, MRI findings, such as tumor size, fluid signal intensity, post-contrast enhancement pattern, and mean apparent diffusion coefficient values of the solid portion, may not be useful parameters for differentiating CCC from EC [22]. Manabe et al. reported that the presence of endometrial disease was a key factor in differentiating EC from CCC [23]. Another MRI morphological feature of EC comprised an internal slit in the solid components [23]. Thus far, very few studies have evaluated the usefulness of morphological features in the differential diagnosis of CCC and EC. It is unclear whether reliable predictors for recognizing type I and type II tumors may comprise useful tools to differentiate CCC from EC.

The purpose of this retrospective study was to assess preoperative MRI characteristics useful for distinguishing between CCC and EC.

## Methods

### Study cohort

This study was approved by the institutional review board of Nara Medical University. Written informed consent was obtained from each patient. The inclusion criteria were as follows: the medical records of patients with CCC and EC who underwent surgical treatment at Nara Medical University Hospital between January 2008 and September 2018 were reviewed. Tumor staging I-IV was performed in accordance with the FIGO classification. A baseline MRI scan was obtained for all patients before any intervention. Contrasted MRI scans were performed less than 2 months before surgery. Patients were excluded from the study if they had no pathology, other pathological subtype of epithelial ovarian cancer, and/or salvage treatment for recurrence and metastatic ovarian cancer at study initiation. One radiologist and one gynecologic oncologist with 10–20 years of experience independently reviewed the MR images of all patients; these reviewers were blinded to the clinical and histopathologic data. This study included 98 patients who met the inclusion and exclusion criteria (primary CCC (*n* = 52) and EC (*n* = 46)).

### Data collection

Data were collected from all enrolled patients and were compared between the two groups. Assessments included baseline characteristics such as age, BMI, parity, and menopausal status.

### Imaging technique

MRI scans were performed on a 3.0 Tesla system (Magnetom Verio; Siemens Healthcare, Erlangen, Germany) with a 32-element body array coil. The protocol of our MRI examination was performed as described previously [24]. MRI scans were T1-weighted, T2-weighted, T1-weighted with fat suppression and gadolinium-enhanced, and DW.

### Imaging features

The following MRI morphologic features were assessed with both T1 and T2 phases, such as the maximal diameter of the cyst, tumor margin, multilocularity, growth patterns and surface irregularities of mural nodules, characteristics of solid components, and the presence of malignant ascites; all of these features are considered significant factors in distinguishing between benign and malignant ovarian tumors [23, 24]. A mural nodule was defined as a wall-based lesion projecting into the cystic space. Several variables involving mural nodules were selected in this study. As described previously, we defined the size of a mural nodule according to its “Height” and “Width” [24]. The term “Height” indicated maximum vertical length from the bottom of the cyst to the top of the nodule. The term “Width” indicated maximum perpendicular length to the “Height.” For each case, the largest dimensions of mural nodules were used to determine “Height” and “Width.” Mural nodular HWR was also calculated. The appearances of tumor margins were classified into two types: a well-defined margin (clear-cut margin) or an ill-defined margin (uncertain margin). The growth pattern of the mural nodules or solid components was designated as one of two categories. The mural nodules were either broad-based nodular structures or polypoid structures. When more than three solid components existed along the inner cystic surface, the pattern was defined as “multifocal, concentric, or broad-based nodular structures” [23]. If one or two mural nodules existed on the inner surface of the cyst, the pattern was defined as “focal, eccentric, or polypoid structures.” When the mural nodules arose from more than one-third of the tumor wall, the pattern was defined as “continuing.” The surface irregularity pattern of the mural nodules or solid components was also divided into two categories: a smooth or regular surface; or a rough, irregular, or lobulated surface. The degree of ascites was graded by using standard criteria, as either negative or positive.

### Statistical analyses

Statistical analyses were performed using JMP software (version 5.0; SAS Institute Inc., Cary, NC, USA). Independent samples t-test (normality) or Mann–Whitney U test (non-normality) methods were used to compare variables between CCC and EC. Variables that were significant in univariate analyses (*p* < 0.05) were used in the multivariate logistic regression model. Survival analyses were performed using the Kaplan–Meier method and log-rank test.

## Results

### Patient demographic factors and tumor characteristic factors

Patient demographic factors of the study population are summarized in Table 1. All patients were of Japanese ethnicity. There were no significant differences between the CCC and EC groups in variables such as age, body mass index (BMI), parity, menopausal status, clinical stage, or International Federation of Gynecology and Obstetrics (FIGO) stage (postsurgical stage). The imaging findings associated with tumor characteristics on MRI were compared between the two groups via univariate analysis (Table 2). There were no significant differences between the CCC and EC groups in variables such as the maximum diameter of the cyst, ill-defined margin, or locularity.

**Table 1:** Patient characteristics of the two groups

	CCC (n = 52)	EC (*n* = 46)	*p* value
Age at diagnosis			
Mean ± SD	56.05 ± 11.62	55.76 ± 10.26	0.894
Median (range)	55.0 (36–90)	54.5 (31–77)	
BMI (kg/m^2^)			
Mean ± SD	22.46 ± 3.69	22.76 ± 4.32	0.713
Median (range)	22.0 (15.4–32.8)	21.9 (11.7–32.4)	
Parity			
Multipara	37	28	0.282
Nullipara	15	18	
Menopausal status			
Premenopause	19	15	0.683
Postmenopause	33	31	
clinical stage			
I / II	42	33	0.265
III / IV	10	13	
FIGO stage (postsurgical stage)			
I / II	43	34	0.290
III / IV	9	12	

*BMI* body mass index, *FIGO* International Federation of Gynecology and Obstetrics, *SD* standard deviation, *CCC* clear cell carcinoma, *EC* endometrioid carcinoma

**Table 2:** Fisher’s exact test for univariate analysis of the two groups

	CCC	EC	OR	95% CI	*p* value
Maximum diameter of the cyst					
< 17.8 (cm)	45	34	0.48	0.162–1.350	0.171
≥ 17.8 (cm)	7	11			
Height of mural nodule					
< 4.0 (cm)	37	27	0.55	0.219–1.336	0.188
≥ 4.0 (cm)	12	16			
Width of mural nodule					
< 5.8 (cm)	41	22	0.20	0.074–0.520	0.0013
≥ 5.8 (cm)	8	21			
HWR					
< 0.69	14	26	3.95	1.665–9.760	0.0022
≤ 0.69	34	16			
Growth pattern of mural nodules					
Focal or eccentric	30	4	0.07	0.019–0.204	< 0.0001
Multifocal or concentric	22	42			
Continuity of mural nodules					
Negative	36	17	0.26	0.110–0.594	0.0017
Positive	16	29			
Appearance of mural nodule margins					
Smooth	18	5	0.23	0.070–0.645	0.0083
Not smooth	34	41			
Absence of ascites					
Negative	33	17	0.34	0.146–0.759	0.0097
Positive	19	29			

*CCC* clear cell carcinoma, *EC* endometrioid carcinoma, *OR* odds ratio, *CI* confidence interval

On univariate analysis, six variables, such as the “Width,” “Height-to-Width” ratio (HWR), growth pattern, and surface irregularity of mural nodules, as well as the presence of ascites and continuity of mural nodules, were significant factors for prediction of CCC. The mural nodule “Width” in patients with EC was statistically significant, compared with that width in patients with CCC (5.42 ± 2.14 cm versus 3.91 ± 1.93 cm, *p* = 0.0013). The patients with EC, when compared with those with CCC, had a smaller HWR (0.69 ± 0.41 cm versus 0.91 ± 0.50 cm, *p* = 0.0022). The multifocal, concentric, or broad-based nodular structures were observed in 29 (63.0%) of 46 patients with EC and 16 (30.7%) of 52 patients with CCC (*p* = 0.0017). Compared with CCC, the mural nodule was significantly wider and more multifocal in EC. Twenty-nine patients (63.0%) with EC showed a continuing mural nodule pattern, whereas 16 patients (38.1%) with CCC showed the continuing pattern (p = 0.0017). Among the 98 patients with EAOC, 50 were classified as “negative” and 48 as “positive,” respectively. Among all patients, 36.5% (19/52) and 63.0% (29/46) had ascites in CCC and EC, respectively (*p* = 0.0097). A round and polypoid mass with high HWR, as well as the absence of ascites, were findings significantly associated with CCC.

**Fig. 1:**
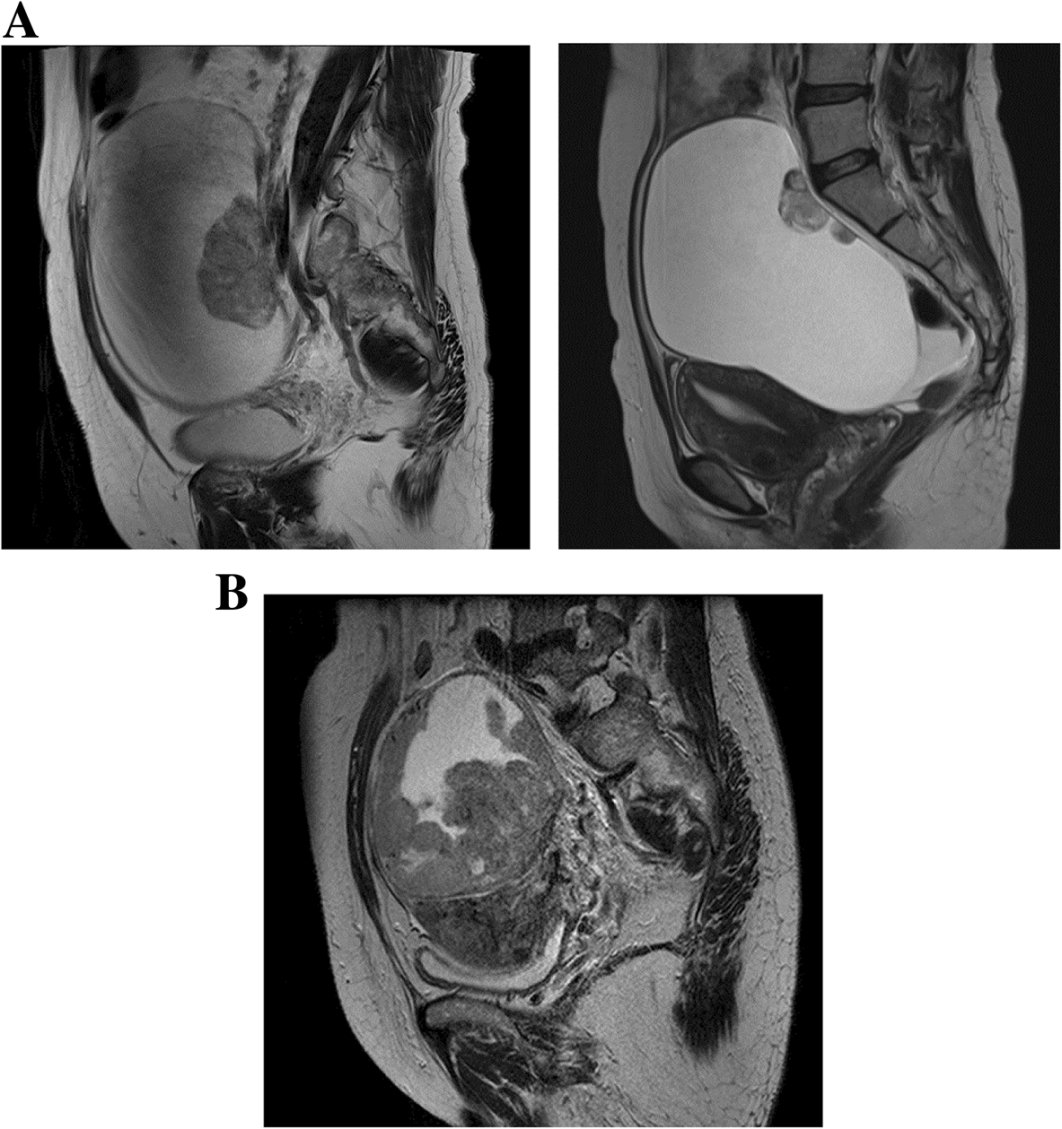
MRI features typical of CCC (**a**) and EC (**b**) lesions. T2-weighted MRI shows examples of CCC mural nodules with a focal, eccentric, or polypoid structure pattern (**a**) and EC mural nodules with a multifocal, concentric, or broad-based nodular pattern (**b**). The multifocal or concentric pattern shows the continuity of each mural nodule. MRI, magnetic resonance imaging; CCC, clear cell carcinoma; EC, endometrioid carcinoma

Figure 1 shows the typical imaging appearances of CCC (A) and EC (B). MRI diagnosis of CCC and EC comprised a large unilocular or multilocular cystic mass associated with several mural nodules protruding into the cystic space. The following imaging characteristics may be aid in diagnosis of CCC: a large cystic mass with a small mural nodule, representing a focal, eccentric, or polypoid growth pattern. In contrast, EC mural nodules exhibited a large, heterogenous mixed mass, representing a multifocal, concentric, or broad-based nodular growth pattern.

### Multivariate logistic regression model

All variables showing significant values in the univariate analysis were included in the multivariate analysis. Multivariate logistic regression analysis revealed that HWR and the growth pattern of the mural nodules resulted in the best discrimination of patients with CCC from those with EC (Table 3). Receiver operating characteristic (ROC) curves showed that the area under the curve (AUC), 95% confidence interval (CI), optimum diagnostic cutoff value, sensitivity, specificity, positive predictive value (PPV), and negative predictive value (NPV) predicted CCC. The AUC for HWR was 0.706 (95% CI: 0.263–0.609), followed by 0.677 (95% CI: 0.286–0.769) for “Width” of a mural nodule (Table 4). The focal growth pattern yielded a sensitivity of 91.3% (95% CI, 82.4–96.4%), specificity of 57.7% (95% CI, 49.8–62.2%), PPV of 65.6% (95% CI, 59.2–69.3%), and NPV of 88.2% (95% CI 76.1–95.1%). The HWR yielded a sensitivity of 70.8% (95% CI, 61.3–79.1%), specificity of 61.9% (95% CI, 51.0–71.4%), PPV of 68.0% (95% CI, 58.8–76.0%), and NPV of 65.0% (95% CI 53.5–74.9%). The focal growth pattern of mural nodules demonstrated a higher HWR for diagnostic sensitivity and improved predictive values for negative test results.

**Table 3:** Multivariate logistic regression analysis for the prediction of clear cell carcinoma

	Standard error	Wald	OR	95% CI	*p* value
Width of mural nodule	0.353	0.325	0.67	0.165–2.695	0.568
HWR	0.312	4.422	3.71	1.128–13.438	0.036
Growth pattern of mural nodule	0.380	12.399	0.69	0.013–0.273	0.0004
Continuity of mural nodules	0.345	0.320	0.68	0.173–2.665	0.572
Appearance of mural nodule margins	0.386	0.085	0.80	0.170–3.720	0.770
Absence of ascites	0.286	0.393	0.70	0.229–2.197	0.531

*OR* odds ratio, *CI* confidence interval, *HWR* Height-to-Width ratio

**Table 4:** Discriminative value of each parameter

	AUC	95% CI	*p* value	cutoff value	sensitivity	specificity	PPV	NPV
Maximum diameter of the cyst	0.504	−0.690-1.265	0.573	17.8 (cm)	0.244	0.865	61.1	57.0
Height of mural nodule	0.540	−0.738-1.268	0.611	4.0 (cm)	0.372	0.755	57.1	57.8
Width of mural nodule	0.677	0.595–2.788	0.003	5.8 (cm)	0.488	0.837	72.4	65.0
HWR	0.706	−2.001-0.151	0.062	0.69	0.691	0.667	64.4	71.1

*AUC* area under the curve, *CI* confidence interval, *PPV* positive predictive value, *NPV* negative predictive value, *HWR* Height-to-Width ratio

### Survival

The 5-year survival rates of patients with CCC and EC were 89.5 and 76.5% (*p* = 0.381). There were no differences in survival between CCC and EC.

## Discussion

In the present study, we aimed to identify the preoperative MRI characteristics that could aid in distinguishing CCC from EC. Both tumors were a large unilocular or multilocular cystic masses associated with several mural nodules protruding into the cystic space. On univariate analysis, the absence of ascites (*p* = 0.0097), a polypoid mural nodule structure (*p* < 0.0001), a smooth rather than lobulated mural nodule surface (*p* = 0.0083), and high HWR (*p* = 0.0022) served as predictors for differentiating CCC from EC. After multivariate analysis, a polypoid mural nodule structure (*p* = 0.0004) and HWR (*p* = 0.036) remained strong and independent predictors.

First, conventional MRI could aid in distinguishing endometriosis-associated ovarian cancer (EAOC) from HGSC [18]. EAOC rarely showed low-signal intensity on T2-weighted images and typically exhibited enhanced mural nodules [18, 19, 25]. The signal intensity on T1-weighted imaging varies from low to very high in CCC [25] and demonstrates homogeneous iso- or hyperintensity in EC [19], suggesting that MR imaging findings on intratumoral cystic components were similar between CCC and EC. The following characteristics were significantly more common for EC than for HGSC: unilateral, round or oval shape, larger mass, mainly cystic with mural nodules or papillary projections, mild ascites, and synchronous primary cancer of the ovary and endometrium [19]. Furthermore, compared with HGSC, the MRI features of CCC were unilateral, unilocular, oval shape, larger mass, mainly cystic with mural nodules or papillary projections, fewer peritoneal implants, and no or mild ascites [18, 26]. Therefore, these two groups exhibited many similar imaging features.

Second, based on these backgrounds, we aimed to identify preoperative imaging characteristics that could assist in the differential diagnosis between CCC and EC. Many cases of malignant mural nodule have been reported in epithelial ovarian cancer [18, 19, 25]; however, the previous studies rarely focused on the MRI features of CCC and EC. Six variables, such as the “Width,” HWR, growth pattern, and surface irregularity of mural nodules, as well as the presence of ascites and continuity of mural nodules, were significant factors for differentiating CCC from EC, according to the univariate analysis. A previous study examined MRI features in nine patients with CCC; it revealed that approximately 90% of CCC lesions showed focal mural nodules [23], a rate much higher than that observed in our study. We found that CCC mural nodules had more focal, eccentric, and polypoid structures (vs. multifocal, concentric, and broad-based nodular structures) than EC (57.7% vs. 8.2%, *p* < 0.0001). On multivariate analysis, HWR and growth pattern of mural nodule remained a strong and independent predictor. Our data indicated that the MRI findings of CCC and EC often overlapped.

Finally, we briefly discuss why CCC predominantly shows a focal, eccentric, or polypoid structure pattern, whereas EC shows a multifocal, concentric, or broad-based nodular structure pattern in the growth of mural nodules. A previous study demonstrated that gene expression profiling could stratify endometriosis into two molecular subtypes: transcription factor hepatocyte nuclear factor 1-beta (HNF-1β)-positive (hypomethylated) and -negative (hypermethylated) cells [27]. Approximately 40% of benign endometriotic cysts expressed HNF-1β [27]. HNF-1β is specifically upregulated in CCC, but not in EC, suggesting that HNF-1β is a key molecule in endometriosis-associated clear cell carcinogenesis and progression [28]. CCC and adjacent atypical endometriosis had HNF-1β overexpression, while benign endometriosis distant from CCC showed negative immunoreactivity for HNF-1β [29]. HNF-1β has been demonstrated as a positive modulator in the survival and growth of CCC cells [30]. CCC cells that arise from HNF-1β positive pre-malignant endometriotic cells would form lesions with focal, eccentric, and polypoid mural nodule structures. In contrast, EC is characterized by epigenetic changes, including considerable estrogen receptor (ER) expression, and could share common estrogen-dependent oncogenic pathways [29]. Up-regulation of ER expression is commonly shared by benign endometriosis, atypical endometriosis, and EC, which may denote a carcinogenic potential in entire areas of endometriotic lesion; this could explain the synchronous and multifocal growth pattern of EC [29]. Notably, EC may comprise an intratumoral metastasis arising from a primary focal EC. The different molecular profiles observed here are likely to contribute to the predominant focal pattern of mural nodule growth in CCC, while EC exhibits a multifocal pattern.

This study had several limitations. First, it was a retrospective study; therefore, there may have been some selection bias in the patients included in the analyses. Second, imaging features were evaluated in a limited number of patients, all of whom were of Japanese ethnicity.

## Conclusions

Here, we revealed that the MRI findings of CCC and EC often overlapped; however, morphological features (e.g., a round mural nodule with high HWR and a focal growth pattern) are useful to distinguish CCC from EC. These potential features may aid clinicians in effective diagnosis. Because this study included only Japanese patients, our conclusions may not apply to patients of other ethnicities. Much work is needed to explore new approaches with high sensitivity and specificity for discriminating CCC and EC.

## Abbreviations

AUC: Area under the curve
BMI: Body mass index
CCC: Clear cell carcinoma
CI: Confidence interval
DW: Diffusion-weighted
EAOC: Endometriosis-associated ovarian cancer
EC: Endometrioid carcinoma
FIGO: International Federation of Gynecology and Obstetrics
HGSC: High-grade serous carcinoma
HWR: Height-to-Width ratio
MRI: Magnetic resonance imaging
NPV: Negative predictive value
OR: Odds ratio
PPV: Positive predictive value
